# Social and Emotional Questionnaire: understanding the social cognition in Brazilian people with Alzheimer Disease through the discrepancy analysis

**DOI:** 10.1002/alz70858_099760

**Published:** 2025-12-25

**Authors:** Tatiana Belfort, Marcela Moreira Lima Nogueira, Isabel Lacerda, Maria Alice Baptista, Michelle Brandt, Rogeria Rangel, Julia Gaigher, Natalie Souza, Marcia C. N. Dourado

**Affiliations:** ^1^ Federal University of Rio de Janeiro, Rio de Janeiro, Rio de Janeiro, Brazil; ^2^ Federal University of Rio de Janeiro, Rio de Janeiro, Brazil

## Abstract

**Background:**

The lack of objective tools to assess social cognition can lead to significant challenges in comparing data from multiple studies. This lack of standardization can make it particularly difficult to understand tasks for individuals with cognitive impairments, such as Alzheimer's disease (AD). Therefore, employing a more straightforward scale that investigates both the perspective of individuals with AD and their informants may provide less biased data when studying this population. The aim of our study was to investigate the pattern of social cognitive impairment using the Social and Emotional Questionnaire (SEQ) in people with AD, differentiating between early‐onset (YOAD) and late‐onset (LOAD) cases, as well as considering different stages of the disease (mild and moderate).

**Method:**

We conducted two cross‐sectional studies involving individuals with AD and their informants. The first study focused on age at onset, including 48 individuals with YOAD and 118 with LOAD. The second study was organized by disease stage, with 87 participants in the mild stage and 50 in the moderate stage. We assessed several aspects, including social cognition, global cognition, quality of life, functioning, neuropsychiatric symptoms, and caregiver burden.

**Result:**

The YOAD group exhibited greater impairment in general cognition, reported poorer quality of life, and experienced more neuropsychiatric symptoms compared to the LOAD group. However, social cognition did not show the same degree of decline and did not differ significantly when compared with informant reports or with individuals with LOAD. A similar trend was observed when analyzing disease stages; while the moderate AD group demonstrated an overall decline compared to the mild group, there were no significant differences in social cognition between these two groups.

**Conclusion:**

By investigating the different perspectives, the social and emotional questionnaire allows us to have a closer understanding of the real functioning of social cognition in people with AD. The results indicate a different progression of social cognition impairment, which leads us to infer that more direct and spontaneous tasks may be associated with automatic processes and appear more stable, whether in a moderately advanced stage of the disease or in a more aggressive progression, such as YOAD.

